# Genome-wide analysis and comparison of the DNA-binding one zinc finger gene family in diploid and tetraploid cotton (*Gossypium*)

**DOI:** 10.1371/journal.pone.0235317

**Published:** 2020-06-29

**Authors:** YuZhe Li, Zhen Liu, KaiYue Zhang, SenYi Chen, MengJie Liu, QingDe Zhang

**Affiliations:** 1 School of Biotechnology and Food Science, Anyang Institute of Technology, Anyang, Henan, China; 2 School of Life Sciences, Henan University, Kaifeng, Henan, China; Brigham Young University, UNITED STATES

## Abstract

The Dof (DNA-binding one zinc finger) transcription factor family is a representative of plant-specific classes of transcription factors. In this study, we performed a genome-wide screening and characterization of the Dof gene family within two tetraploid species *Gossypium barbadense*, *Gossypium hirsutum*, and two diploid species *Gossypium arboreum*, *Gossypium raimondii*. 115, 116, 55 and 56 Dof genes were identified respectively and all of the genes contain a sequence encoding the Dof DNA-binding domain. Those genes were unevenly distributed across 13/26 chromosomes of the cotton. Genome comparison revealed that segmental duplication may have played crucial roles in the expansion of the cotton Dof gene family, and tandem duplication also played a minor role. Analysis of RNA-Seq data indicated that cotton Dof gene expression levels varied across different tissues and in response to different abiotic stress. Overall, our results could provide valuable information for better understanding the evolution of cotton Dof genes, and lays a foundation for future investigation in cotton.

## Introduction

Transcription factors play a very vital role in gene regulation at transcriptional level. The Dof (DNA-binding one zinc finger) is a plant-specific transcription factor having multiple roles such as carbon assimilation, light-mediated regulation, seed maturation and germination [1]. Dof specifically bind AAAG sequences of plant gene promoters with the Dof DNA-binding domain [2–4]. In spite of high level homology in the Dof domain, the rest of the sequences are divergent, coinciding with their expected diverse functions [1, 3].

Cotton (*Gossypium*) is one of the most important agronomic genera in the world. Furthermore, cotton is also an excellent model system for studying polyploidization and cell elongation [5–8]. Current understanding recognizes more than 50 species within the cotton genus, with both diploid and polyploid members. Cotton is commonly grouped into eight diploid genomic groups, designated A-G and K, and one tetraploid genomic group, namely AD. All tetraploid cotton species came from interspecific hybridization between the A-genome species and the D-genome species [9, 10].

In recent years, as more and more plant genome data have been published, genome-wide analysis has become an very effective way for gene function prediction from a large family of genes [11, 12] and there are an increasing number of reports about cotton gene families [13–17]. The Dof gene family has been extensively studied in many plant species, such as *Arabidopsis thaliana*, *Oryza sativa* [18], *Jatropha curcas* [19] and *Setaria italic* [20]. Furthermore, the Dof gene family of *Gossypium hirsutum* was also studied [21, 22]. Because of the importance of Dof gene in various physiological processes, it would be necessary to perform a genome-wide identification and comparative analysis of Dof family in different cotton species. Whole genome sequenes of two cultivated tetraploid species, upland cotton (*Gossypium hirsutum*) and island cotton (*Gossypium barbadense*) [23], and two diploid species *Gossypium arboretum* [24] and *Gossypium raimondii* [25] provided an opportunity to reveal the traits of cotton Dof gene family at genome-wide level. In the present study, we performed a comprehensive analysis of cotton Dof genes, including their gene structure, motif compositions, chromosome distribution, duplication patterns and expression profiles. This study will provide valuable clues for functional characterization of Dof gene family in cotton.

## Materials and methods

### Identification and characterization of the cotton Dof genes

The *G*. *hirsutum* [23] and *G*. *barbadense* [23] genome sequences were downloaded from CottonGen (https://www.cottongen.org/), The genome sequences of *G*. *arboretum* [24] were downloaded from NCBI (BioProject ID: PRJNA382310), and the *G*. *raimondii* [25]genome sequence was download from https://cottonfgd.org/. The candidate genes were searched by BLASTP [26] using a E value of 1e-10 and the known Dof proteins from Arabidopsis were taken as queries. Then the hidden Markova model file (PF02701) was downloaded from the Pfam website (http://pfam.xfam.org/) and was used as the query to identify all possible Dof sequences with HMMER software [27]. Furthermore, NCBI CD-Search (https://www.ncbi.nlm.nih.gov/cdd/) and Search Pfam tools (http://pfam.xfam.org/search) were used to confirm the candidate sequence. The biophysical properties of the Dof proteins were calculated using the ExPASy online server tool (https://www.expasy.org/).

### Phylogenetic and gene structure analysis of Dof proteins

Previous studies have shown that there are 36 Dof proteins in *Arabidopsis thaliana* [18]. In this study, we included these *Arabidopsis thaliana* Dof proteins in the phylogenetic tree. The ClustalX [28] was used to align Dof protein sequences and MEGA-X [29] program was used to construct a neighbor-joining phylogenetic tree with 1000 bootstrap replicates. Dof gene sequences were loaded into TBtools (http://www.tbtools.com/) to obtain exon-intron structure. To identify protein-conserved motifs of cotton Dof, a MEME [30] search was performed, the maximum number of motif was set to 10.

### Chromosomal localization, synteny analysis and gene duplication of cotton Dof genes

The chromosome locations of all Dof genes were obtained from the genome annotation files of four cotton species and Mapchart [31] was used to visually map the chromosomal location. Gene duplication events were analyzed using MCScanX [32] and the result data were plotted by TBtools. Thereafter, the synonymous (Ks) and nonsynonymous (Ka) substitution rates of Dof genes were calculated by KaKs_Calculator 2.0 [33].

### Expression profile analysis in various tissues of cotton Dof genes

The original expression data for *G*. *hirsutum* and *G*. *barbadense* Dof genes of multiple tissues and under salinity, PEG, cold, heat conditions and normal condition (CK) for 1h, 3h, 6h, 12h and 24h were retrieved from NCBI BioProject database (PRJNA490626). The software Trimmomatic [34] was used to remove the adapters and to perform quality control. The program hisat2 [35] was used to map the reads to the genomes, then the expression profile of Dof genes was obtained with FPKM value using Cufflinks [36], then the results were log transformed and a heatmap was generated by MeV [37].

## Results

### Genome-wide identification and characterization of Dof gene family in cotton

We used a whole-genome scan to identify genes that encode proteins containing the Dof domain by both BLASTP and HMMER. In the present study, we identified 115, 116, 55 and 56 Dof genes from *G*. *hirsutum*, *G*. *barbadense*, *G*. *arboreum* and *G*. *raimondii*. The gene number in tetraploid cotton is almost twice that of diploid cotton, and is more than in rice (30 Dof genes) and Arabidopsis (36 Dof genes) [18]. The length of these cotton Dof protein sequences mainly centered on the range of 164~543 amino acid residues. Correspondingly, the molecular weights were mainly distributed from 18318.89 Da to 59589.04 Da. The predicted isoelectric point of Dof proteins varied from 4.77 to 9.92 (S1 Table). The Dof gene family has a wide range of characteristics, this is similar both in cotton and other species [1, 18, 19].

**Fig 1:**
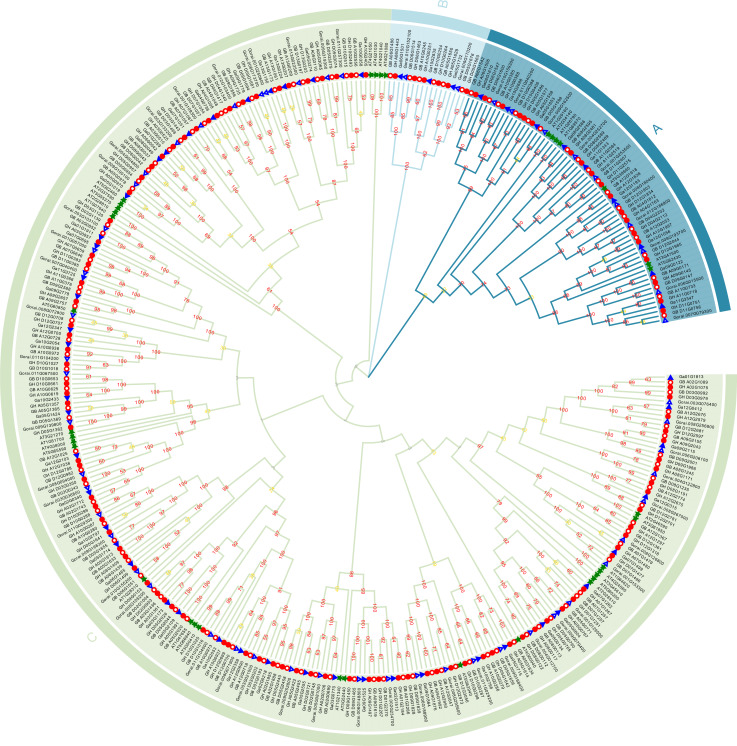
Phylogenetic tree of the Dof gene family. Bootstrapping values are indicated as percentages along the branches. The different background colors indicate different groups. Tetraploid *G*. *hirsutum* is indicated by red solid circle and tetraploid *G*. *barbadense* is indicated by red hollow circle; diploid *G*. *arboreum* is indicated by blue solid triangle and *G*. *raimondii* is indicated by blue hollow triangle; the *Arabidopsis thaliana* is indicated by green star.

The 342 Dof family members were classified into 3 groups: A, B, C (Fig 1), and the genome/subgenomes of each analyzed cotton have similar member number in the 3 groups (Fig 1, S1 Table).

### Gene structure and conserved motifs of the cotton Dof gene family

Our results revealed that the number of exons varied from 1 to 4 in cotton Dof gene family. Most of genes have 1 (43.6%) or 2 (48.8%) exons, and only 1 gene in *G*. *hirsutum* and 1 gene in *G*. *raimondii* contains 4 exons (S1 Fig).

Dof protein usually has a DNA-binding domain of approximate 40~60 amino acid residues in the N-terminus. This domain contains a highly-conserved CX2CX21CX2C single zinc-finger structure, which is essential for the zinc finger configuration and loop stability [1, 3, 4]. In this study, all of the cotton Dof protein sequences were loaded into MEME to identify the conserved motifs. The results show that a total of ten conserved motifs were observed. Among them, motif-1 is a common motif in all cotton Dof proteins, corresponding to the CX2CX21CX2C single zinc-finger structure in the Dof domain (S1 and S2 Figs). Some of the Dof proteins only contain motif-1, while others have extra specific motifs, which may be relevant to different functions.

### Chromosomal locations and gene duplication events of the cotton Dof gene family

**Fig 2:**
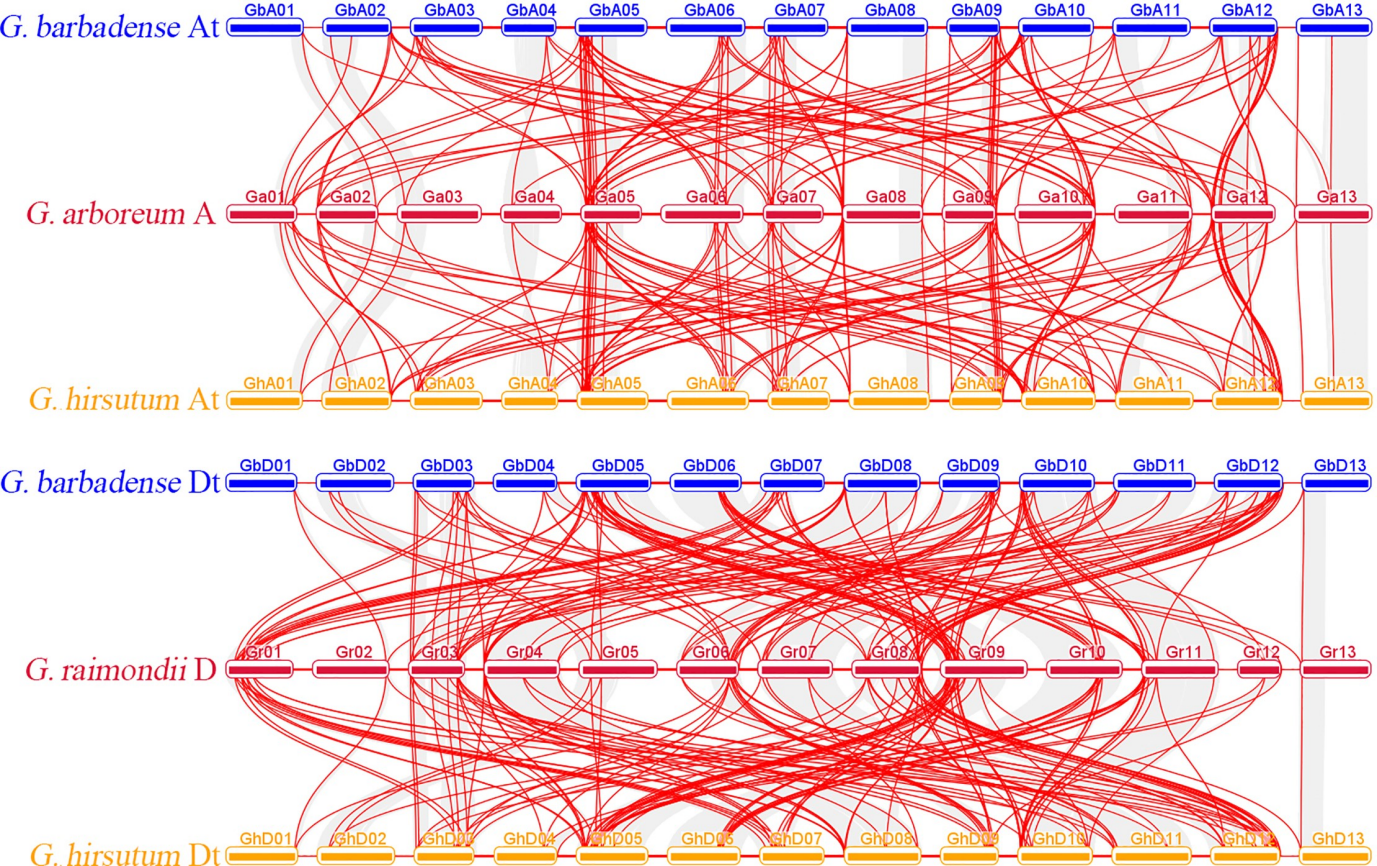
The sub-genome distribution and synteny analysis of cotton Dof genes. The red lines indicate duplicated Dof pairs, the gray lines indicate collinear blocks.

The results show that the 342 cotton Dof genes were widely but unevenly distributed on 13/26 cotton chromosomes (S3 Fig), which is similar to millet [20], banana [34] and Physic Nut [19]. As gene replication plays an important role in the occurrence of novel functions and gene expansion, in this study, we analyzed the duplication events of cotton Dof genes. According to our MCScan analysis, 128, 125 duplication gene-pairs were found between diploid *G*. *arboreum* A-genome and tetraploid *G*. *hirsutum*, *G*. *barbadense* A-subgenome respectively. 137 144 duplication gene-pairs were found between diploid *G*. *raimondii* D-genome and tetraploid *G*. *hirsutum*, *G*. *barbadense* D-subgenome respectively (Fig 2). In addition, we also identified the tandem duplication events. According to Holub, a chromosomal region within 200 kb containing two or more genes is defined as a tandem duplication event [19]. Fourteen Dof genes were clustered into six tandem repeat event regions in both *G*. *hirsutum* and *G*. *barbadense*, six Dof genes were clustered into three tandem repeat event regions in *G*. *arboreum*, and severn Dof genes were clustered into three tandem repeat event regions in *G*. *raimondii*.

To further infer the phylogenetic mechanisms of cotton Dof family, we constructed a collinear maps associated with all of the four cotton species analyzed (S4 Fig). Some collinear pairs were identified between all of the four cotton species, such as GB_A05G1633/GB_D05G1655/Ga05G1714/ GH_A05G1613/GH_D05G1641/Gorai.009G168300, indicating that these orthologous pairs may already exist before the ancestral divergence. In contrast, some collinear gene pairs were not found in one or more of the four cotton species, such as Gorai.003G036800/Ga02G0340/ GB_A02G1743/GB_D03G0343/GH_A02G1712/GH_D03G0350, which may indicate that these orthologous pairs formed after the divergence of the four cotton species (S4 Fig).

**Fig 3:**
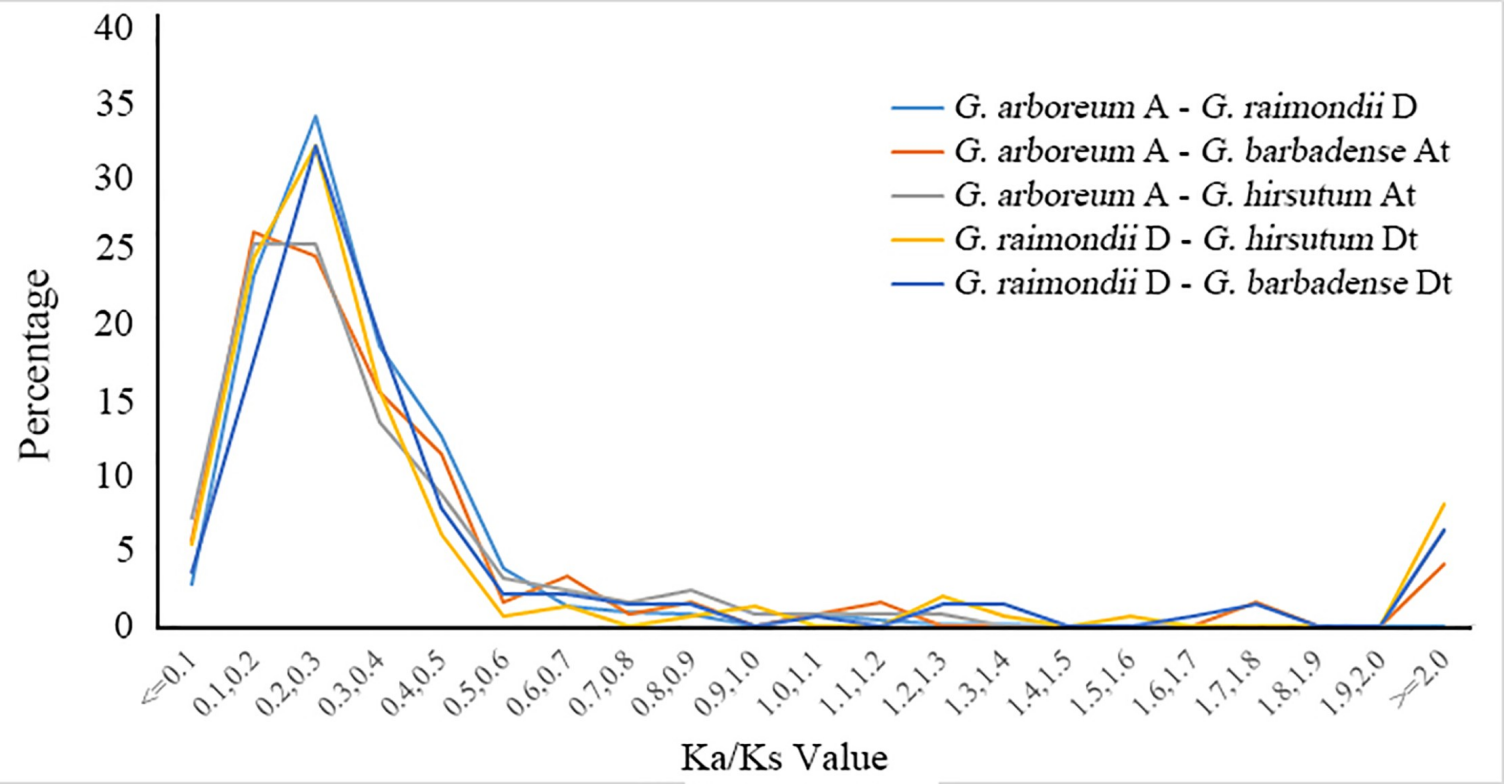
The distribution of Ka/Ks.

We further calculated the Ka/Ks ratios for genes pairs between A/At and D/Dt genomes/subgenomes, and the majority of orthologous Dof gene pairs Ka/Ks ratio were between 0.2 and 0.3 (Fig 3), suggesting that the cotton Dof gene family might have experienced purifying selective pressure during evolution.

### Expression pattern of the Dof genes

**Fig 4:**
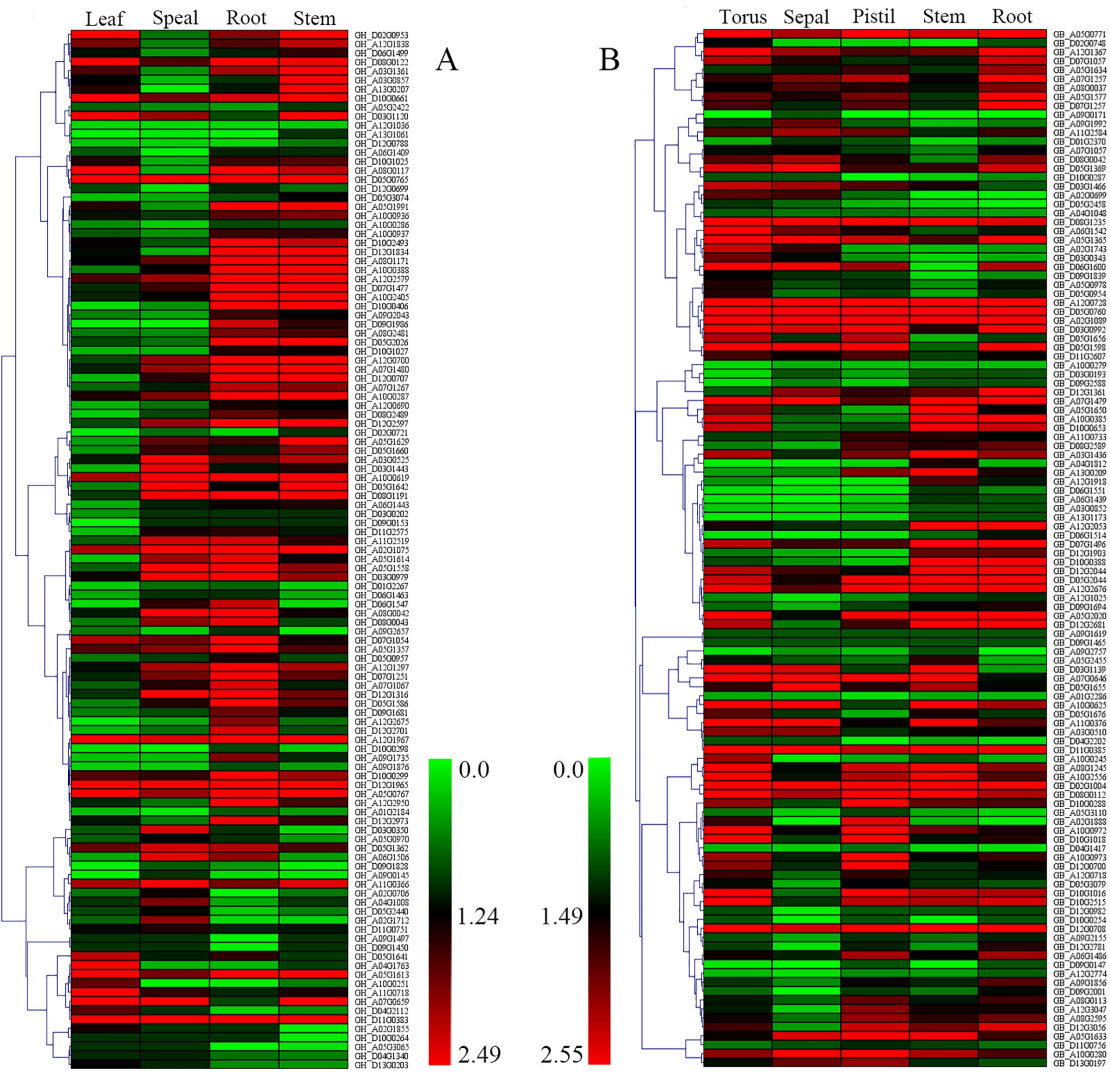
Expression patterns of Dof genes under different tissue. A: *G*. *hirsutum*, B: *G*. *barbadense*.

Transcriptome data was used to explore the variation of cotton Dof genes expression across tissues. RNAseq data for leaf, sepal, root and stem of *G*. *hirsutum* and torus, sepal, pistil, stem and root of *G*. *barbadense* were downloaded and analyzed. Our results show that the expression level of most Dof genes vary greatly in different tissues (Fig 4), which is similar to the expression pattern of millet Dof genes [20]. At the same time, few Dof genes have similar expression in different tissues, for example GH_A12G1967, GH_D12G1965 and GH_D05G0765 expressed highly across all tested tissues, while others, such as GH_D12G0788 and GH_A12G1036, expressed far lower in all tested tissues (Fig 4A). In general, these expression patterns indicate that paralogous Dof genes differ considerably in their biological regulatory functions.

**Fig 5:**
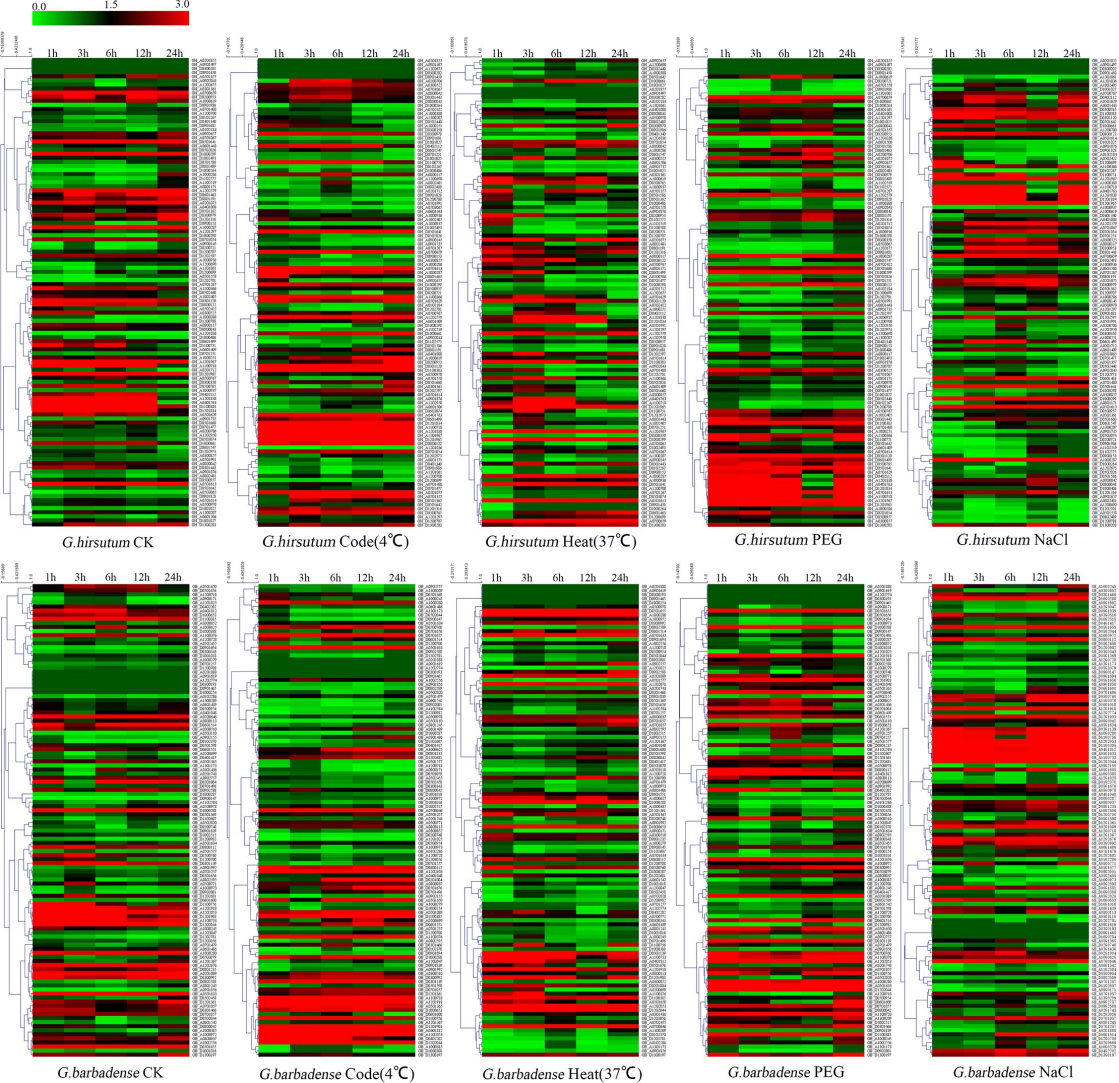
Expression patterns of Dof genes under normal condition (CK), cold, heat, PEG and salinity conditions for 1h, 3h, 6h, 12h and 24h.

To further confirm the potential functions of Dof genes in abiotic stress responses, the expression of *G*. *hirsutum* and *G*. *barbadense* Dof genes under salinity, PEG, cold, heat conditions and normal condition for 1h, 3h, 6h, 12h and 24h was analyzed (Fig 5). GH_D03G0202, GH_A02G1855 and GH_A09G1497 of the 115 *G*. *hirsutum* Dof genes, and GB_D09G1465, GB_A09G1619 and GB_D03G0193 of the 116 *G*. *barbadense* Dof genes were not expressed in all detected samples. Most Dof genes were significantly induced/repressed by multiple treatments. For instance, GH_A11G0718, GH_D11G0751 of *G*. *hirsutum* and GB_A11G0733, GB_D11G0756 of *G*. *barbadense* responded to salinity, PEG, cold and heat treatments significantly. Interestingly, all of these genes were up-regulated by salinity, PEG, cold stress but were down-regulated by heat stress treatment. In addition, GH_D06G1463, GH_A09G2657 of *G*. *hirsutum* and GB_D06G1514 of *G*. *barbadense* were repressed by all tested treatments. In contrast, other Dof genes exhibited preferential expression under different conditions. For instance, GH_D12G1965 of *G*. *hirsutum* and GB_A09G2757 of *G*. *barbadense* were induced significantly by salinity and cold stress but not obviously by heat and PEG stress. Overall, these results demonstrated that the cotton Dof gene family displayed different expression patterns under diverse environmental stress conditions, suggesting that these genes were responsive to stress treatments.

## Discussion

Transcription factors are an important group targeted for crop improvement and a lot of efforts have been made to reveal the whole set of transcription factors [35–37]. In the present study, we completed genome-wide analysis of cotton Dof genes by bioinformatic analysis and 115 and 116 Dof genes were identified from tetraploid *G*. *barbadense* and *G*. *hirsutum*, 55 and 56 Dof genes were identified from diploid *G*. *arboreum* and *G*. *raimondii*. The number of genes in tetraploid cotton is almost twice that of diploid cotton, but the duplication gene-pairs in tetraploid cotton is significantly more than that in diploid cotton. The Dof gene density decreased from 0.3/Mb to 0.05/Mb in Arabidopsis and cottons. The reason for this discrepancy might be the variable status of genome duplications in Arabidopsis and cottons [18, 34, 38]. The cotton Dof family members were classified into 3 groups, the same group shared more similar gene structures which suggest evolutionary conservation in cotton Dof gene evolution. The gene expansion of the Dof family in cotton mainly resulted from segmental duplication, and tandem duplication also played a minor role. The Dof duplicated gene pairs tended to be subjected to positive selection, which may play important roles in the adaptive phenotypes of cotton. In this study, the gene expression of Dof gene family were identified in salinity, PEG, cold, heat conditions and normal condition stresses. The expression profile demonstrated the broad involvement of cotton Dof genes in different abiotic stressed treatments. In addition, cotton Dof gene expression has tissue-specific characteristics.

Because *G*. *hirsutum* is the main source of textile fiber, the study of cotton Dof gene family was focused on *G*. *hirsutum* in previous studies [21, 22]. Studies have shown that *G*. *hirsutum* Dof gene family constitutively expressed in leaves, roots and stems, accumulated highest in leaves. The salinity and cold treatments induced *G*. *hirsutum* Dof transcript accumulation, and the overexpressed of Dof showed significantly higher salt and cold tolerance over the wild-type plants [21]. Moreover, genome-wide study shown that there were 114 Dof genes in *G*. *hirsutum*, the phylogeny, duplication, and chromosomal locations of *G*. *hirsutum* Dof gene family in previous studies are similar to ours [22]. In this study, we performed a genome-wide analysis and comparison of the Dof gene family within two tetraploid cotton species and two diploid cotton species. Gene structure, conserved motifs and Ka/Ks distribution of Dof gene family in the four cotton species were analyzed for the first time. In addition, Dof gene expression was analyzed by RNA-Seq data in our study which is different with RT-PCR in previous studies. Therefore, our study will further broaden our insights into the evolution and functional elucidation of Dof gene family in cotton.

## Conclusions

A genome-wide bioinformatics analysis of cotton Dof genes was performed in this study. Protein lengths, molecular weights, and theoretical isoelectric points of cotton Dofs vary greatly. Gene structure analysis demonstrated that 92.4% cotton Dof genes have 1 or 2 exons. Conserved motif, phylogenetic tree and expression pattern were also analyzed in our study. On the whole, this study provides an extensive resource for understanding the Dof genes in cotton.

## Supporting information

S1 Table Classification and characterization of cotton Dof genes. (XLSX)

S1 Fig Phylogenetic relationships, gene structure and architecture of conserved protein motifs in Dof genes. The motifs, numbers 1~10, are displayed in different colored boxes. The sequence length can be estimated using the scale at the bottom.
(EPS)

S2 Fig Sequence logo of the cotton Dof protein conserved motif1. The font size represents the frequency of the respective amino acid.
(TIF)

S3 Fig Chromosomal locations of cotton Dof genes. (EPS)

S4 Fig The chromosomal distribution and interchromosomal relationships of cotton Dof genes. The lines indicate duplicated Dof pairs.
(TIF)
