# Supplementary figures and images for: Genome-wide analysis and comparison of the DNA-binding one zinc finger gene family in diploid and tetraploid cotton (Gossypium)
Source: PLoS One. 2020 Jun 29;15(6):e0235317. doi: 10.1371/journal.pone.0235317 (PMC7323982; doi:10.1371/journal.pone.0235317)

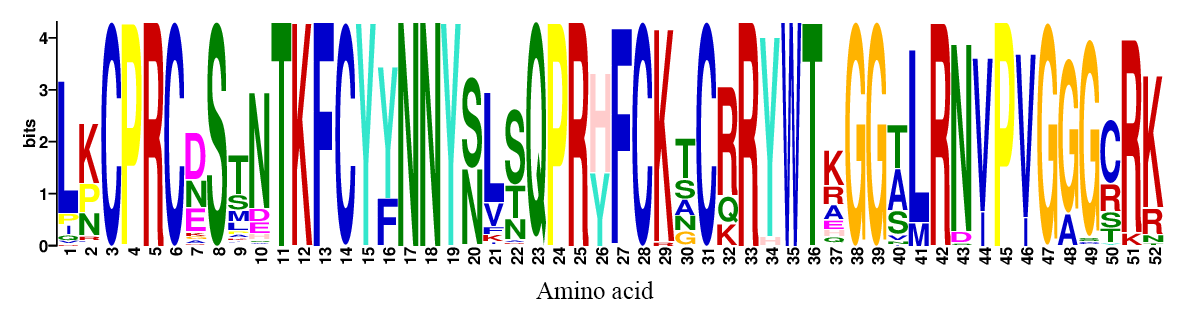

Supplement: S2 Fig — The font size represents the frequency of the respective amino acid. (TIF) [file pone.0235317.s003.tif]

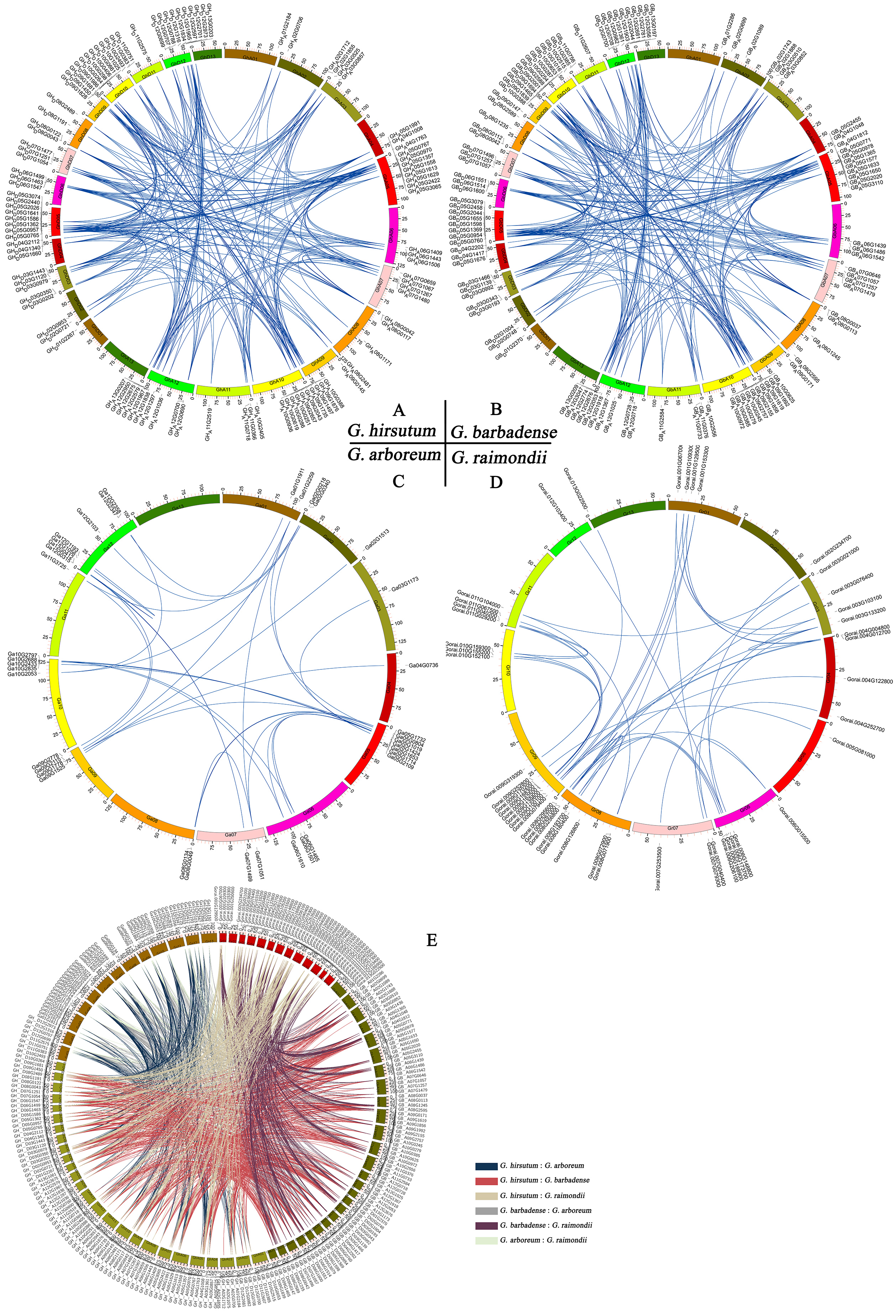

Supplement: S4 Fig — The lines indicate duplicated Dof pairs. (TIF) [file pone.0235317.s005.tif]
